# A336C/A336T/T337C variations in HBV core gene and spontaneous hepatitis B e antigen loss in chronic hepatitis B patients

**DOI:** 10.1186/1743-422X-8-226

**Published:** 2011-05-14

**Authors:** Wen Fan, Lu Huang, Zhiming zhou, Yirong Li

**Affiliations:** 1 Laboratory Department, Union Hospital, Tongji Medical College, Huazhong University of Science and Technology, Wuhan, China 430022; 2 Laboratory Department, Jingzhou First People's Hospital, Jingzhou, China 434000; 3 Laboratory Department, West Area of Union Hospital, Tongji Medical College, Huazhong University of Science and Technology, Wuhan, China 430065

## Abstract

**Background:**

A336C/A336T/T337C variations in HBV core gene were demonstrated to relate to the decreases in serum HBV DNA levels and HBV replication in chronic hepatitis B patients. Usually the drastic decrease in serum HBV DNA levels correlates with spontaneous HBeAg loss during the course of chronic HBV infection. The aim of the present study was to investigate whether there was correlation between A336C/A336T/T337C variations and spontaneous HBeAg loss

**Methodology/Principal Findings:**

A modified PCR-RFLP assay and ELISA were adopted to determine A336C/A336T/T337C variations and serum HBeAg levels in chronic hepatitis B patients without any antiviral therapy, respectively, whereas G1896A variation and HBV genotype were detected using Taqman-PCR assay. RFLP pattern C, E, G, C/G mixture and a new pattern C' were found in this study. A336C/A336T/T337C variations occurred in 40/166(24.1%) chronic hepatitis B patients. Chi-square test showed that C336/T336/C337 variants was more frequent in chronic hepatitis B patients with A1896 variants than those with the wild type G1896 (χ2 = 4.7, P = 0.03), and moreover, patients with C336/T336/C337 variants had a significantly lower HBeAg-positive percentage than those with the wild type A336/T337. Binary logistic regression identified genotype B (OR = 4.1, 95%CI = 1.8-9.2, P = 0.001), the presence of C336/T336/C337 variants (OR = 3.2, 95%CI = 1.2-8.5, P = 0.02) and A1896 variants (OR = 7.8, 95%CI = 3.3-18.5, P < 0.001) as independent factors associated with spontaneous HBeAg loss.

**Conclusion/Significance:**

A336C/A336T/T337C were naturally occurring polymorphisms in HBV core gene, and moreover, the presence of C336/T336/C337 variants was first demonstrated to be an independent factor associating with spontaneous HBeAg loss in chronic hepatitis B patients.

## Background

Infection with the hepatitis B virus (HBV) remains a very important human disease, with an estimated 400 million people infected chronically worldwide. It is reported that about 1.5 million people die annually as a result of infection, most of them in Asia and Africa. In China, chronic HBV infection is also a major public health problem and associated with a wide range of clinical states, from an asymptomatic carrier state with a normal liver function to severe liver disease, which includes liver cirrhosis and hepatocellular carcinoma[1,2].

Spontaneous hepatitis B e antigen (HBeAg) loss during the course of chronic HBV infection is usually accompanied by the disappearance of the biochemical markers of hepatitis and a drastic decrease in viral replication. Factors associated with a higher rate of spontaneous HBeAg loss include certain HBV genotypes and certain HBV genetic diversities. Currently, HBV is classified into eight genotypes (A-H) based on an intergroup divergence of 8% or more in the complete nucleotide sequence of viral genomes. Genotypes B and C are prevalent in China. Previous cross-sectional and retrospective studies showed that genotype B patients had a significantly lower prevalence of HBeAg at presentation and a significantly higher rate of spontaneous HBeAg loss than genotype C[3-6]. In addition to genotype B, G1896A variation, not A1762T/G1764A dual variations, is proposed to be another major mechanism to explain spontaneous HBeAg loss in the natural history of chronic HBV infection[7,8]. G1896A variation was mainly present in patients who were HBeAg-negative natives of Asian or Mediterranean basin, whereas A1762T/G1764A dual variations were detected in a similar proportion of HBeAg-negative and HBeAg-positive patients[6,9-12].

A336C/A336T/T337C variations in HBV core gene, destroying the cleavage sites of *Tsp*509I, were demonstrated to correlate with the decreases in serum HBV DNA levels and HBV replication in chronic hepatitis B patients, and moreover, A336C/A336T variations caused the substitution of Glu-83 with Asp in HBcAg[13]. HBeAg is transcribed from the precore/core gene like HBcAg, therefore, A336C/A336T variations also cause the change of amino acid composition of HBeAg. Recent studies showed HBeAg up-regulation resulted in the decrease in TLR levels, which protected HBV from the immune response and led to higher HBV DNA viral load in HBeAg positive patients[14,15]. In addition, one multicenter study found that HBeAg titre correlated positively with serum HBV DNA and HBV replication[16]. Therefore, it was reasonable to suspect that A336C/A336T/T337C variations caused the decrease in serum HBV DNA levels by down-regulating HBeAg expression or promoting HBeAg loss. In order to investigate whether there was correlation between A336C/A336T/T337C variations and spontaneous HBeAg loss, the cumulative rates of spontaneous HBeAg loss between patients with C336/T336/C337 variants and the wild type A336/T337 were compared by analyzing a cohort of 166 Chinese chronic hepatitis B patients without any antiviral therapy.

## Methods

### Patients, samples and the extraction of HBV DNA

This is a cross-sectional study using stored serum samples from 166 chronic hepatitis B patients with genotype B and genotype C in Union Hospital of Huazhong University of Science and Technology in 2009. Chronic HBV infection was defined as persistent positivity for hepatitis B surface antigen (HBsAg) for at least six months at presentation. Patients with malignancy, liver cirrhosis, anti-HCV positivity, anti-HIV positivity and autoimmune disease were excluded. Before collecting blood samples, all patients had never been treated with any antiviral therapy. Informed consent was obtained from all of the patients. The study was approved by the Ethics Committee of Union Hospital, Tongji Medical College, Huazhong University of Science and Technology. Serum samples were stored at -80°C before they were tested. HBV DNA was extracted from 100 μl of serum with extraction solution in a PCR-fluorescence quantification kit for HBV (Kelong, Shanghai, China) by the protocol described by the manufacturer.

### Serology

HBsAg, HBeAg, anti-HBs antibodies, anti-HBc antibodies, anti-HBe antibodies, anti-HCV and anti-HIV antibodies were determined with commercial assay kits (InTec, Xiamen, China).

### Real-time quantification of serum HBV-DNA

Quantification of serum HBV-DNA was carried out by real-time PCR assay using the method described before[13]. HBV-DNA levels were expressed as log copies/ml.

### HBV core gene specific PCR-RFLP (polymerase chain reaction-restriction fragment length polymorphism) assay and HBV core gene sequencing

The presence of HBV core gene A336C/A336T/T337C variations in patient's sera were determined by a modified PCR-RFLP assay and cleaved PCR products were electrophoresed on 2.5% agarose gel[13,17], and five PCR amplicons per RFLP pattern were sequenced directly to confirm the accuracy of PCR-RFLP assay. The restriction enzyme *Tsp*509I and primers for amplification and sequencing were in agreement with those used in reference[13].

### Detection of G1896A variation in HBV core gene

G1896A variation in HBV core gene was detected with PCR-fluorescence detection kit based on Taqman MGB (minor groove binder) probes (Biocore, Hangzhou, China). The amplification and detection were carried out using the LightCycler^® ^Systems for real-time PCR (Roche Diagnostic, Switzerland). For each sample, real-time PCR reaction A and B were performed in two different capillary tubes according to the instructions of the manufacturer. In brief, the real-time PCR reaction was carried out in a final volume of 20.4 μl, consisting of 18.4 μl of master reaction mixture A or B and 2 μl of DNA template. Thermal cycling conditions were as follows: One cycle of 37°C for two minutes and 94°C for two minutes, followed by 35 cycles of 93°C for five seconds and 62°C for 30 seconds at a programmed temperature transition rate of 20°C/s, then cooled at 40°C for less than one second. Monitoring of fluorescence intensity occurred at regular intervals during the annealing-extension phase. The recombinant plasmids including wild type G1896(or A1896 mutant) were used as negative control(or positive control). Ct value obtained from real-time PCR was adopted to identify wild type G1896 and A1896 mutant and the identification standards were list in Table 1.

**Table 1:** The standards for identifying HBV genotypes, G1896 and A1896

	Identification standards
A1896	Ct(A)≤38 and Ct(B)≥40
G1896	Ct(A)≤38 and Ct(B)≤38
Genotype B	Ct(FAM)≤36 and Ct(Hex)≥40
Genotype C	Ct(FAM)≥40 and Ct(Hex)≤36
Genotype B/C mixture	Ct(FAM)≤36 and Ct(Hex)≤36
Uncertain Genotype	Ct(FAM)≥40 and Ct(Hex)≥40

### HBV genotyping

HBV was genotyped with PCR-fluorescence diagnosis kit based on Taqman probes (Kelong, Shanghai, China). The amplification and detection were carried out with the MXP3000^® ^real-time thermocycler (Stratagene, La Jolla, CA, USA). Real-time PCR reaction was performed in eppendorf tube according to the instruction provided by the manufacturer. In brief, the real-time PCR reaction was carried out in a final volume of 30 μl, consisting of 26 μl of master reaction mixture and 4 μl of DNA template. Real time PCR program consisted of heating at 50°C for 2 minutes and 94°C for 5 minutes, followed by 40 cycles of a two stage temperature profile of 93°C for 15 seconds and 60°C for 45 seconds at a programmed temperature transition rate of 3°C/s. Fluorescence emitted from reporter dyes FAM and Hex™ was monitored at regular intervals during the annealing-extension phase respectively. Ct value obtained from real-time PCR was adopted to identify HBV genotypes and the identification standards were list in Table 1.

### Statistical analysis

Statistical analysis was carried out with the SPSS 15.0 software package. Chi-square test was used for comparison of categorical data. Binary logistic regression analysis was adopted to estimate the independent factors associating with spontaneous HBeAg loss. During statistical analysis, serum ALT(alanine aminotransferase) levels, serum HBV DNA levels and age were transformed into categorical variables, respectively: (1) serum ALT levels were categorized into normal group(≤50 U/L) and abnormal group (>50 U/L); (2) serum HBV DNA levels were categorized into mild elevated group (<6.0) and severely elevated group(≧6.0); (iii)age were grouped into <35 years and ≧35 years. The results were presented as odds ratios(OR), 95% confidence intervals (CI), wald values and P values. P ≤ 0.05 was considered statistically significant.

## Results

### RFLP patterns and A336C/A336T/T337C variations in chronic hepatitis B patients

**Figure 1:**
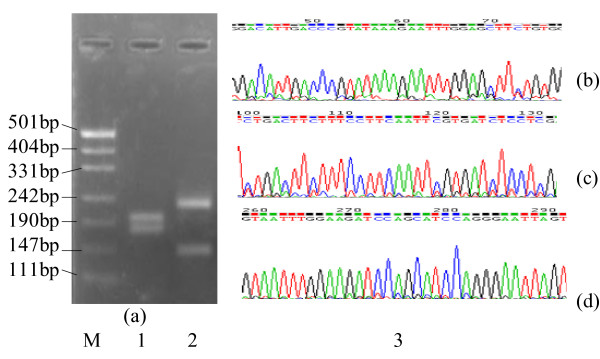
**HBV core gene RFLP pattern C'(M. DNA marker;1. RFLP E;2. RFLP C' 3**. Cleavage site of *Tsp509I *were found at position 61(b),116(c),261 and 286(d) corresponding to nucleotide 110,165,310 and 335 in HBV core gene showing RFLP C', respectively).

A336C/A336T/T337C variations in HBV core gene were determined with a modified PCR-RFLP assay on total 166 serum samples collected from chronic hepatitis B patients. Five RFLP patterns cleaved by restriction enzyme *Tsp*509I, namely RFLP C, C', E, G and C/G mixture were found. RFLP C' was a new pattern found in this study(see Figure 1). RFLP C, C', E, G and C/G mixture occurred in 101(60.8%),1(0.006%) 24(14.5%),27(16.3%),13(7.8%) samples, respectively. Sequencing of HBV core gene demonstrated complete match between RFLP patterns and SNPs (single nucleotide polymorphisms) A336C/A336T/T337C (data not shown). Sequencing also showed that A336C/A336T/T337C variations occurred in serum samples presenting with RFLP patterns G and C/G mixture (A336C/A336T/T337C variations were found in part of HBV DNA from serum samples with RFLP patterns C/G mixture, therefore, serum samples showing RFLP C/G mixture were also considered to be presence of C336/T336/C337 variants). A336C/A336T/T337C variations occurred in 40/166(24.1%) chronic hepatitis B patients without any antiviral therapy (See Table 2).

**Table 2:** Comparison of important characteristics between 166 patients with the wild type A336/T337 and A336C/A336T/T337C variants

	A336/T337 (n = 126)	C336/T336/C337 (n = 40)	χ2	p value
HBeAg			12.1	0.001
Positive	68	9		
Negative	58	31		
G1896 variation			4.7	0.03
G1896	75	16		
A1896	51	24		
HBV genotype			3.4	0.07
B	61	26		
C	65	14		
serum HBV DNA levels				
<6	69	31	6.6	0.01
≥6	57	9		
Age				
<35	62	18	0.2	0.64
≥35	64	22		
Sex			2.4	0.12
Male	100	27		
Female	26	13		
Serum ALT levels			0.1	0.74
≤50 U/L	75	25		
>50 U/L	51	15		

### A336C/A336T/T337C variations correlated with G1896A variation and the decrease in serum HBV DNA levels

G1896A was the most common variation in precore gene and occurred in 75/166(45.2%) chronic hepatitis B patients in this study. C336/T336/C337 variants were found in 24/75 (32.0%) chronic hepatitis B patients with A1896 variants and 16/91(17.6%) chronic hepatitis B patients with the wild type G1896. Chi-square test showed C336/T336/C337 variants were more frequent in chronic hepatitis B patients with A1896 variants than those with the wild type G1896 (χ2 = 4.7, P = 0.03), suggesting that A336C/A336T/T337C variations correlated closely with G1896A variation (See Table 2). In addition, patients with C336/T336/C337 variants had a significant lower rate of severely elevated serum HBV DNA levels than those with the wild type A336/T337 (χ2 = 6.6, P = 0.01).

HBV genotype was determined on total of 166 chronic hepatitis B patient with Taqman-PCR assay. Of the 166 patients, 87 (52.4%) were infected with HBV genotype B and the remaining 79 (47.6%) were infected with genotype C. A336C/A336T/T337C variations occurred in 26/87 (29.9%) chronic hepatitis B patients with genotype B and 14/79 (17.7%) patients with genotype C, and Chi-square test showed that there was no significant difference in the rate of A336C/A336T/T337C variations between HBV genotype B and C (χ2 = 3.3, P = 0.07, See Table 2).

### The presence of C336/T336/C337 variants was an independent factor associating with spontaneous HBeAg loss in chronic Hepatitis B patients

In this study, HBeAg was found in 77/166(46.4%) chronic hepatitis B patients. Chi-square test showed chronic hepatitis B patients with C336/T336/C337 variants had a significantly lower HBeAg-positive percentage than those with the wild type A336/T337(22.5% vs 54.0%, χ2 = 12.1, P = 0.001), indicating that the presence of C336/T336/C337 variants was closely associated with spontaneous HBeAg loss in chronic hepatitis B patients (See Table 3). Furthermore, Chi-square test also showed that the presence of A1896 variant, age ≧35 years, HBV genotype B and serum HBV DNA levels <6.0 were also related closely to spontaneous HBeAg loss in chronic hepatitis B patients (See Table 3), therefore, A336C/A336T/T337C variations, serum HBV DNA levels, G1896A variation, age and HBV genotype were entered into a binary logistic regression model to determine which was the independent factor associating with spontaneous HBeAg loss. The results of the analysis were displayed in Table 4. Although the presence of A1896 variant (OR = 7.8, 95% CI = 3.3-18.5, P < 0.001) and HBV genotype B(OR = 4.1, 95% CI = 1.8-9.2, P = 0.001) were the most two importantly independent factors associating with spontaneous HBeAg loss, the presence of C336/T336/C337 variants was also an independent factor associating with spontaneous HBeAg loss(OR = 3.2, 95% CI = 1.2-8.5, P = 0.02) (See Table 4).

**Table 3:** Comparison of important characteristics between 166 patients with positive HBeAg and negative HBeAg

	HBeAg positive (n = 77)	HBeAg negative (n = 89)	χ2	P value
A336C/A336T/T337C variations			12.1	0.001
A336/T337	68	58		
C336/T336/C337	9	31		
G1896 variation			42.3	<0.001
G1896	63	28		
A1896	14	61		
HBV genotype			14.8	<0.001
B	28	59		
C	49	30		
serum HBV DNA levels			23.9	<0.001
<6	31	69		
≥6	46	20		
Age			4.6	0.03
<35	44	36		
≥35	33	53		
Sex			0.001	0.94
Male	59	68		
Female	18	21		
Serum ALT levels			3.2	0.07
≤50 U/L	52	48		
>50 U/L	25	41		

**Table 4:** Binary logistic regression analysis of potential factors associating with spontaneous HBeAg loss

Factors	Odds Ration	95%CI	Wald value	P value
A336C/A336T/T337C variations				
A336/T337	1			
C336/T336/C337	3.2	1.2-8.5	5.2	0.02
G1896 variation				
G1896	1			
A1896	7.8	3.3-18.5	21.4	<0.001
HBV genotype				
B	4.1	1.8-9.2	11.8	0.001
C	1			
serum HBV DNA levels				
<6	1			
≧6	0.5	0.2-1.1	3.3	0.07
Age				
<35	1			
≧35	1.9	0.8-4.1	2.4	0.12

## Discussion

Recent studies have identified several HBV variants and mutants playing an important role in disease pathogenesis, immune escape, and resistance to antiviral therapy[18,19]. HBV variants such as A1896 occur under the selective pressures from the host's immune system and from viral factors [20,21], whereas HBV mutants, such as rtI204 and rtV204, usually occur secondary to exogenous factors such as lamivudine and associate with a specific phenotype[12,22]. In this cross-sectional study, 166 serum samples from chronic hepatitis B patients without any antiviral therapy were enrolled, and A336C/A336T/T337C variations and G1896A variation occurred in 24.1% (40/166) and 45.2% (75/166) serum samples, respectively. Statistical analysis showed A336C/A336T/T337C variations were closely associated with G1896A variation and more prone to occur in samples with A1896 variants, therefore, A336C/A336T/T337C were naturally occurring polymorphisms as well as G1896A. In addition, chronic hepatitis B patients with C336/T336/C337 variants had a significant lower rate of severely elevated serum HBV DNA levels than those with the wild type A336/T337, which was in agreement with previous report that A336C/A336T/T337C variations caused the decreases in serum HBV DNA levels and HBV replication[13].

The natural history of chronic HBV infection can be divided into four phases: immune tolerance, HBeAg-positive chronic hepatitis, inactive HBsAg carrier and HBeAg-negative chronic hepatitis[23]. The common features of immune tolerance phase and HBeAg-positive chronic hepatitis phase are positive for HBeAg and high levels of serum HBV DNA, whereas inactive HBsAg carrier phase is characterized by the absence of HBeAg and decrease in HBV DNA. Therefore, spontaneous HBeAg loss during the course of chronic HBV infection usually correlates with a drastic decrease in serum HBV DNA and disappearance of biochemical markers of hepatitis[23-27]. In this study, the rate of severely elevated serum HBV DNA levels in HBeAg-positive patients was higher than that in HBeAg-negative patients, supporting that HBeAg expression was strongly related to HBV replication and high serum HBV DNA levels. However, binary regression analysis showed that serum HBV DNA levels <6.0 was not an independent factor associating with spontaneous HBeAg loss. One retrospective and follow-up study carried out in Taiwan showed that rates of spontaneous HBeAg loss were not statistically different between patients with low and high baseline serum HBV DNA levels and baseline serum HBV DNA levels were not independent factors for predicting HBeAg loss[5]. Taken together, these findings did not support that serum HBV DNA levels were independent factors associating with spontaneous HBeAg loss in chronic hepatitis B.

HBeAg loss can occur spontaneously or secondary to treatment with nucleoside/nucleotide analogs and interferon. Spontaneous HBeAg loss, which occurs annually in as many as 10% to 20% of those with HBeAg-positive hepatitis, is an important landmark in the natural history of chronic HBV infection. In a population-based study of 1536 Alaskan natives who had HBV infection in adulthood, spontaneous loss was found in 70% during the 10-year follow-up period[28]. Factors associated with a higher rate of spontaneous HBeAg loss include the presence of A1896 variant and genotype B[5,23,29]. One cross-sectional study from China found that among 223 HBV DNA-positive chronic patients, the G1896A variation was found in 47/128 HBeAg-negative patients and 5/95 HBeAg-positive patients[3]. Similarly, in another multicenter study of 449 chronic hepatitis B patients with detectable HBV DNA, the A1896 variant was present in 7% of HBeAg-positive and 36% of HBeAg-negative patients[11]. This study showed A1896 variant was present in 14/77 (18.2%) HBeAg-positive patients and 61/89 (68.5%) HBeAg-negative patients. Furthermore, binary regression analysis showed patients who presented with A1896 variant, as compared to having wild type G1896, were nearly 7.8 times more likely to occur spontaneous HBeAg loss (OR = 7.8, 95% CI = 3.3-18.5, P < 0.001), which supporting the presence of A1896 variants was one of the major mechanisms to explain spontaneous HBeAg loss in the natural history of chronic HBV infection.

In this study, genotypes B and C accounted for 52.4% (87/166) and 47.6% (79/166) of chronic hepatitis B patients respectively. HBV genotype B was thought to be related to spontaneous HBeAg loss. One retrospective study of 146 Taiwanese adult HBeAg-positive hepatitis B carriers showed genotype C patients had a significantly lower rate of spontaneous HBeAg loss than genotype B patients[5]. Another retrospective study of 332 Chinese patients with a mean follow-up of 48 months, genotype B patients had a significantly lower prevalence of HBeAg at presentation and a significantly higher rate of spontaneous HBeAg loss during follow-up[3]. In agreement with the above two reports, chronic patients with genotype B were demonstrated to have a higher likelihood of spontaneous HBeAg loss than those with genotype C (OR = 4.1, 95% CI = 1.8-9.2, P = 0.001) in this study. It was more interesting to find that chronic hepatitis B patients with C336/T336/C337 variants had a significantly lower HBeAg-positive percentage than those with the wild type A336/T337, and moreover, logistic regression analysis showed the likelihood of HBeAg loss among chronic hepatitis B patients with C336/T336/C337 variants was nearly 3.2 times that of those with the wild type A336/T337 (OR = 3.2, 95% CI = 1.2-8.5, P = 0.02), which demonstrated that the presence of C336/T336/C337 variants was an independent factor associating with spontaneous HBeAg loss. Therefore, A336C/A336T/T337C variations were proposed to be another major mechanism to explain spontaneous HBeAg loss in the natural history of chronic HBV infection as well as G1896A variation and genotype B[7,8,30,31]. Age was thought of as another factor influencing spontaneous HBeAg loss, but the real significance of age is now existence controversy. One long-term follow-up study in Alaska showed that older HBV carriers were more likely than younger carriers to clear HBeAg[28]. The other follow-up study in Taiwan showed age ≤35 years was an independent factor associating with spontaneous HBeAg loss[5]. In this study chronic hepatitis B patients were grouped into age <35 years and age ≧35 years, and HBeAg-positive percentage were higher in group age <35 years than that in group age ≧35 years, but binary regression analysis in this study showed that age ≧35 years were not an independent factor associating with spontaneous HBeAg loss (OR = 1.8, 95% CI = 0.8-4.1, P = 0.12), which was not agreement with one of the above two studies. The possible reason was below: (i) cross-sectional study was used in this study, whereas retrospective and follow-up studies were adopted in the above two studies; (ii) age was a dynamic increasing variable.

In conclusion, A336C/A336T/T337C were naturally occurring polymorphisms in HBV core gene, and the presence of C336/T336/C337 variants was first demonstrated to be an independent factor associating with spontaneous HBeAg loss. This study also demonstrated that the presence of A1896 variant and HBV genotype B were also independent factors related to spontaneous HBeAg loss in chronic hepatitis B patients.

## Competing interests

The authors declare that they have no competing interests.

## Authors' contributions

WF performed most of the experimental work. LH participated in specific PCR-RFLP assay. ZZ participated in the detection of G1896A variation in HBV core gene. YL was responsible for the planning of the study, data analysis, and drafted the manuscript. All authors have read and approved the final manuscript.
